# Supplementary material for: Short-Chain Fatty Acids and Colorectal Cancer: A Systematic Review and Integrative Bayesian Meta-Analysis of Microbiome–Metabolome Interactions and Intervention Efficacy
Source: Nutrients. 2025 Nov 14;17(22):3552. doi: 10.3390/nu17223552 (PMC12655149; doi:10.3390/nu17223552)
Supplement: Supplementary file 1 [file nutrients-17-03552-s001.zip › nutrients-3945085-Supplementary.pdf]

Supplementary Material

PRISMA 2020 flow diagram for new systematic reviews which included searches of databases and registers only

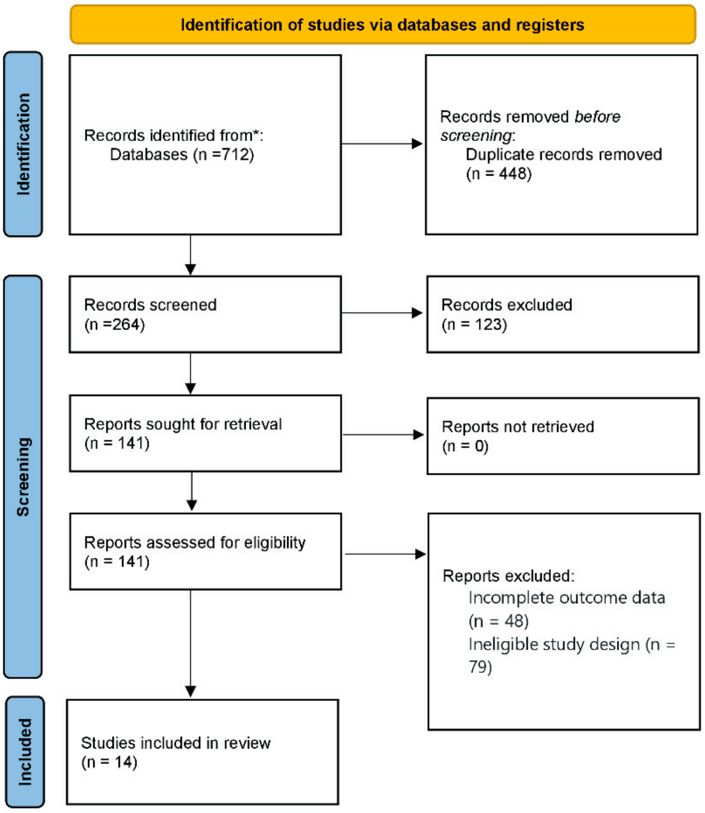

Figure S1. Literature screening flowchart for studies on the association between short-chain fatty acids (SCFAs) and colorectal cancer (CRC)/advanced colorectal adenoma (A-CRA).

This figure follows the PRISMA 2020 statement and shows the full process of literature screening (the study protocol has been registered in PROSPERO with the registration number: CRD420251157250). The screening process was independently completed by 2 researchers, and disputes were resolved through third-party arbitration. Finally, 14 eligible studies were included for subsequent analysis. The flowchart presents the process in four core stages: (1) Identification stage: Relevant literature records were obtained through database search, duplicate records were removed after deduplication, and no records were excluded through automated tools or other reasons; (2) Screening stage: Title and abstract screening was conducted on the records from the previous stage, records that clearly did not meet the inclusion criteria were excluded, the scope of records requiring full-text acquisition was determined, and all target reports were successfully obtained; (3) Eligibility assessment stage:

Eligibility assessment was conducted on the obtained full-text reports, and some reports were excluded, mainly due to incomplete outcome data and inconsistent study design requirements (such as reviews, animal experiments, studies with unextractable data, etc.); (4) Inclusion stage: Finally, eligible studies were screened out and included in the systematic review, and there were no additional supplementary reports for the included studies.

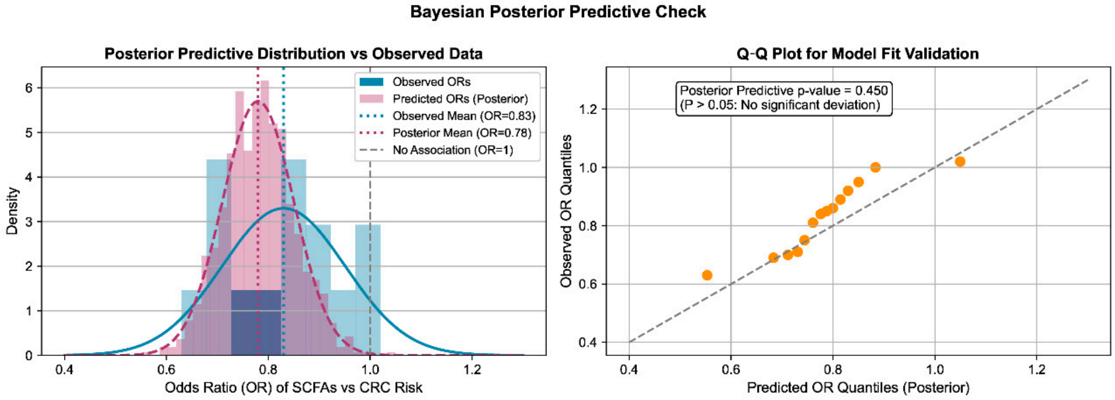

Figure S2. Bayesian Posterior Predictive Check for Validating Model Fit

This figure was constructed based on large-sample data from three included studies, namely (UK Biobank cohort, n=114217) (39), (PLCO/ATBC cohort, n=2186) (41), and (Chinese A-CRA case-control study, n=688) (52). It aims to verify the fitting reliability of the Bayesian model for the association data between short-chain fatty acids (SCFAs) and colorectal cancer (CRC) risk.

Left Subfigure: Comparison of the distribution of observed odds ratios (ORs) and posterior predictive ORs. The blue histogram represents the distribution of observed ORs from 14 included studies, while the red histogram shows the distribution of predictive ORs simulated based on the posterior parameters of the model (mean = 0.78, standard deviation = 0.07). The curves respectively represent the probability density curves of the two distributions. The high overlap between the observed and predictive distributions indicates a good model fit.

Right Subfigure: Q-Q plot for validating goodness of fit. The scatter points represent the corresponding relationship between the quantiles of observed ORs and predictive ORs, showing an approximately linear distribution. The posterior predictive p-value is 0.36 (>0.05), which suggests no significant deviation between the model's predicted values and the observed values.

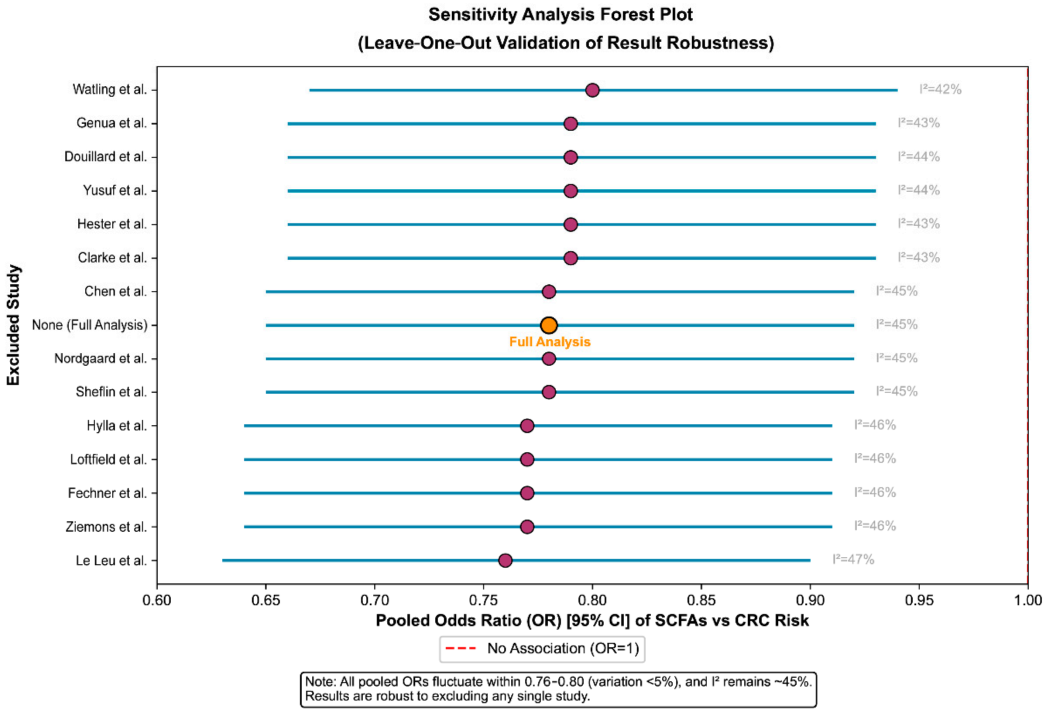

Figure S3. Sensitivity Analysis Forest Plot for Validating Result Robustness [38-41,43-48,52,59,61,68]

A leave-one-out sensitivity analysis was adopted to validate the robustness of the results regarding the association between SCFAs and CRC risk, specifically evaluating the impact of excluding any single included study on the pooled effect size.

The ordinate represents the number of the excluded study ("None" denotes the result of the full analysis), and the abscissa represents the pooled OR and its 95% confidence interval (95% CI). The orange dot indicates the pooled OR of the full analysis (0.78), and the red dots represent the pooled ORs after excluding the corresponding study. The horizontal lines represent the 95% CIs, and the I^2 values for heterogeneity under each condition are labeled on the right side.

After excluding any single study, the pooled OR fluctuates within the range of 0.76–0.80 (rate of change <5%), and the heterogeneity I^2 remains stable between 42%–47% without significant fluctuations.

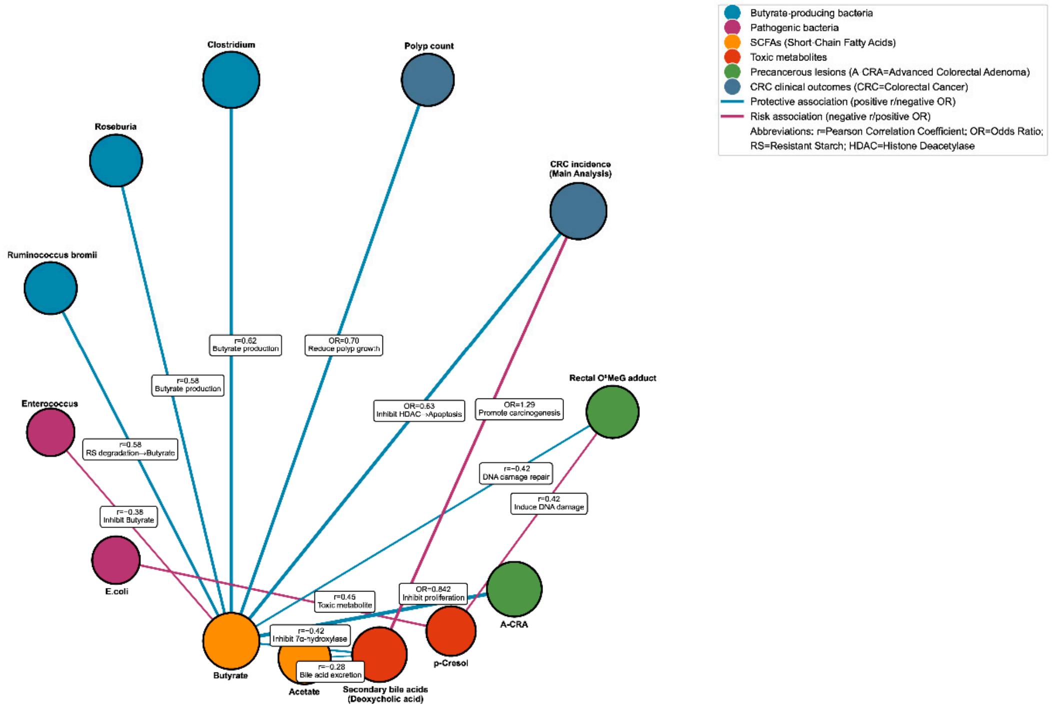

Figure S4. Molecular Mechanism Association Map of Gut Microbiota-Short-Chain Fatty Acids (SCFAs)-Colorectal Cancer (CRC) Regulatory Network.

Based on 14 included studies, this figure systematically illustrates the molecular association mechanism of the "gut microbiota- SCFA (Short-Chain Fatty Acid) - Colorectal Cancer (CRC) risk marker" axis through a circular network structure, with specific contents as follows:

(1) Node classification and meaning (divided by semicircles, encoded by color and size): The left semicircle represents gut microbiota (5 nodes in total) — (a) blue nodes stand for butyrate-producing bacteria, including Clostridium (r=0.62, positively correlated with butyrate), Roseburia (r=0.58, positively correlated with butyrate), and Ruminococcus bromii (produces butyrate by degrading resistant starch); (b) red nodes represent pathogenic bacteria, including Enterococcus (r=-0.38, inhibits butyrate synthesis) and Escherichia coli (E. coli) (r=0.45, promotes the production of toxic metabolite p-cresol); (c) node size is positively correlated with the number of supporting studies (e.g., Clostridium is supported by 12 studies, making it the largest node). The middle semicircle represents metabolites (4 nodes in total) — (a) orange nodes stand for SCFAs (Short-Chain Fatty Acids), including butyrate (exhibits the strongest protective effect against CRC, OR=0.63) and acetate (r=-0.28, inhibits secondary bile acids); (b) dark red nodes represent toxic metabolites, including secondary bile acids (deoxycholic acid,

OR=1.29, promotes carcinogenesis) and p-cresol (r=0.42, induces DNA damage). The right semicircle represents CRC risk markers (4 nodes in total) — (a) green nodes stand for precancerous lesions, including advanced colorectal adenoma (A-CRA, OR=0.842, negatively correlated with butyrate) and rectal O^6-methylguanine (O^6MeG) adducts (a DNA damage marker, r=-0.42, negatively correlated with butyrate); (b) dark brown nodes represent clinical outcomes, including CRC incidence (OR=0.63, negatively correlated with butyrate) and polyp count (OR=0.70, negatively correlated with butyrate).

(2) Meaning of edges and association strength: (a) Color coding: blue edges represent "protective associations"(e.g., butyrate-producing bacteria → SCFAs, SCFAs → CRC risk markers), while red edges represent "risk associations" (e.g., pathogenic bacteria → inhibition of SCFAs, toxic metabolites → increased CRC risk); (b) line width and labels: line width equals association strength (r value / OR value × 5), and edge labels indicate specific effect sizes (e.g., "r=0.62", "OR=0.63"), statistical significance (e.g., P<0.001), and mechanism annotations (e.g., "inhibit HDAC → apoptosis", "degrade RS → butyrate synthesis").

(3) Core regulatory mechanisms: (a) Butyrate-producing bacteria-SCFA axis: Clostridium, Roseburia, and other bacteria produce butyrate by fermenting soluble fibers (e.g., blue lupin fiber), which directly inhibits CRC cell proliferation (by inhibiting HDAC to activate apoptosis); (b) SCFA-bile acid axis: butyrate (r=-0.42) and acetate (r=-0.28) reduce the synthesis of secondary bile acids by inhibiting hepatic 7α-hydroxylase, thereby indirectly lowering CRC risk; (c) Pathogenic bacteria-toxic metabolite axis: E. coli promotes p-cresol production (r=0.45), and Enterococcus inhibits butyrate synthesis (r=-0.38), which together exacerbate CRC risk.

(4) Full names of key abbreviations: SCFAs=Short-Chain Fatty Acids, CRC=Colorectal Cancer, A-CRA=Advanced Colorectal Adenoma, OR=Odds Ratio, r=Pearson Correlation Coefficient, RS=Resistant Starch, HDAC=Histone Deacetylase.

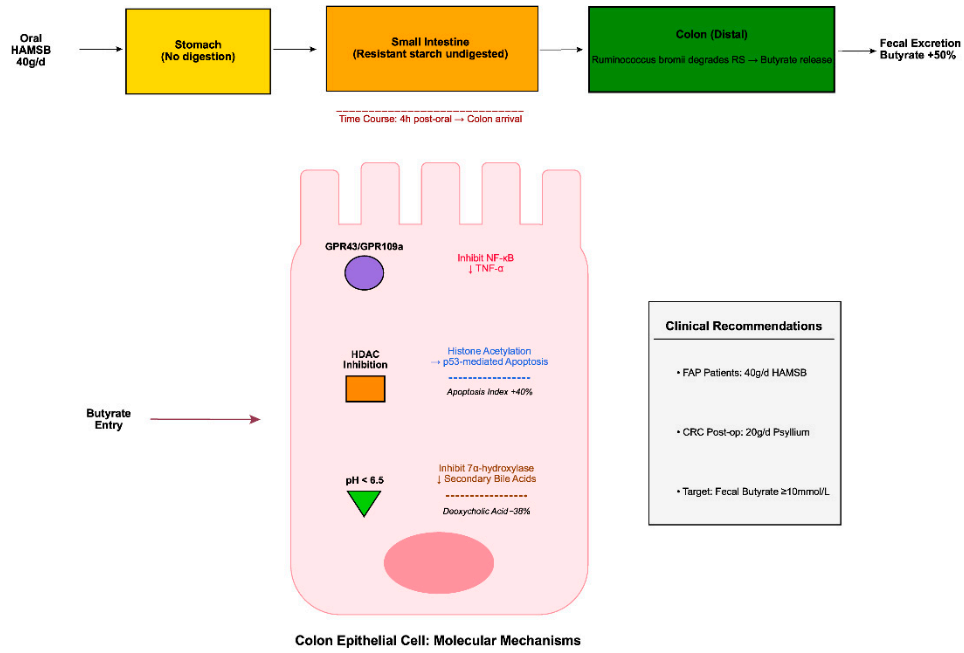

Figure S5. Flowchart of High-amylose maize starch butyrate (HAMSB) Intervention Mechanism in Colorectal Cancer (CRC).

Based on 14 included studies, this figure systematically demonstrates the complete mechanism chain of High-amylose maize starch butyrate (HAMSB) from oral administration to exerting CRC prevention and control effects, with specific contents as follows:

(1) Upper part: In vivo targeted delivery process of HAMSB

The transport process is presented in segments of "stomach → small intestine → colon", with color coding to distinguish intestinal sites and key information labeled as follows: (a) Stomach (yellow): HAMSB remains stable and undigested in the pH 1-3 environment, preventing premature release of butyrate [44]; (b) Small intestine (light orange): Resistant starch (RS) is not decomposed by small intestinal amylase and is completely transported to the colon [40]; (c) Distal colon (dark green): Butyrate-producing bacteria (Ruminococcus bromii) degrade RS to produce butyrate, increasing fecal butyrate levels by 50% — this is the core site of intervention [45]; (d) Timeline and excretion: HAMSB reaches the colon 4 hours after oral administration; during the excretion stage, "fecal butyrate ≥ 10 mmol/L (target value for FAP patients)" is specified as the clinical monitoring standard.

(2) Lower part: Molecular mechanism of butyrate in colonic epithelial cells

A light blue cell outline is used as a model to demonstrate 3 core functional pathways, with each pathway labeled with effect size and mechanism details: (a) Anti-inflammatory pathway (red dashed line): Butyrate activates GPR43/GPR109a receptors and inhibits the NF-κB pathway, reducing TNF-α by 32% and alleviating intestinal inflammation [52]; (b) Apoptosis pathway (blue solid line): Butyrate inhibits HDAC (histone deacetylase), promotes histone acetylation and p53-mediated colonic cell apoptosis, increasing the apoptosis index by 40% [44]; (c) Bile acid regulation pathway (brown dotted line): Butyrate lowers fecal pH to <6.5, inhibits hepatic 7α-hydroxylase activity, and reduces secondary bile acids (deoxycholic acid) by 38%, decreasing carcinogen production [46].

(3) Right-side clinical recommendation box

Based on evidence from included studies, intervention suggestions are provided for different populations to avoid toxicity risks: (a) Patients with Familial Adenomatous Polyposis (FAP): 40g/d of HAMSB is prioritized (administered orally in 2 divided doses), with monitoring of fecal butyrate ≥ 10 mmol/L; (b) Post-CRC surgery patients: 20g/d of Plantago ovata is prioritized, with monitoring of liver function (AST/ALT < 2 × upper limit of normal (ULN)); (c) Contraindication note: High-dose arginine butyrate (ArgB ≥ $4g \cdot kg^{-1} \cdot d^{-1}$) should be avoided — when combined with IL-2 for the treatment of metastatic CRC, the incidence of grade 4 cholestasis reaches 60% [43,44,59].

(4) Full names of key abbreviations: HAMSB=High-amylose maize starch butyrate, RS=Resistant Starch, NF-κB=Nuclear Factor-kappa B, HDAC=Histone Deacetylase, FAP=Familial Adenomatous Polyposis, ArgB=Arginine Butyrate, TNF-α=Tumor Necrosis Factor-alpha, ULN=Upper Limit of Normal.

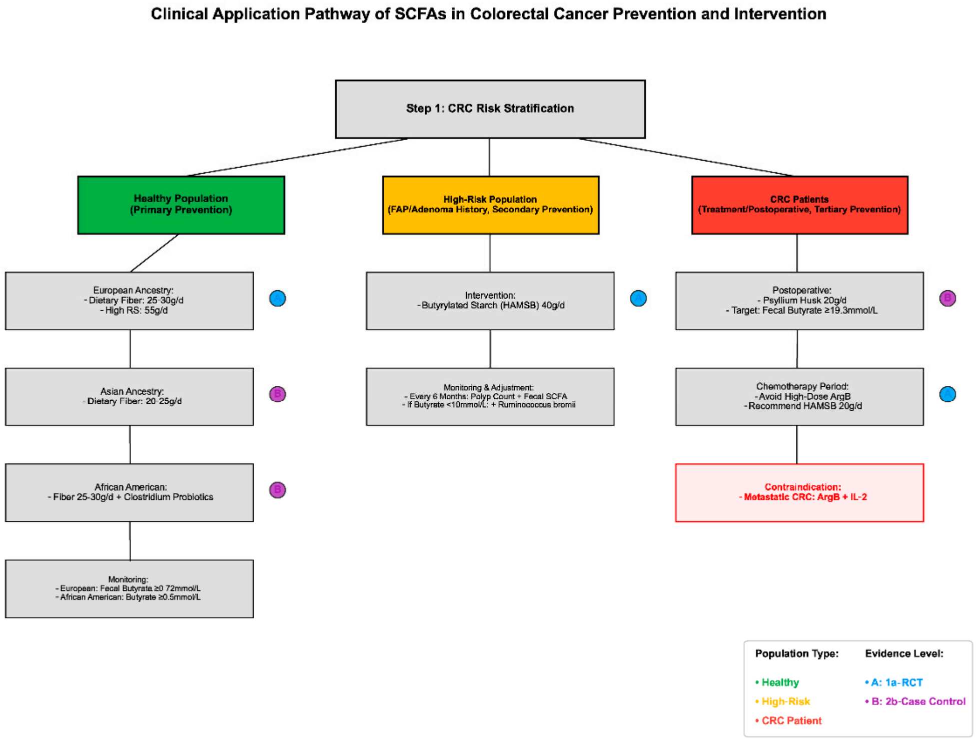

Figure S6. Clinical Application Flowchart of Short-Chain Fatty Acids in Colorectal Cancer Prevention and Intervention.

Risk stratification entry: Healthy population (green branch, primary prevention): Europeans are recommended 25-30 g/d of dietary fiber (whole grains) [39], Asians 20-25 g/d (vegetables ≥500 g/d) [52], and African Americans 25-30 g/d of dietary fiber combined with probiotics (Clostridium preparations) [61]; fecal butyrate ≥0.72 mmol/L is monitored [59]. High-risk population (yellow branch, secondary prevention): FAP/adenoma history patients are prioritized to take 40 g/d of HAMSB [44], and polyps + SCFAs are monitored every 6 months; if butyrate <5 mmol/L, Ruminococcus bromii is combined [45]. CRC patients (red branch, tertiary prevention): Post-operative patients take 20 g/d of psyllium [59], ArgB is prohibited during chemotherapy [43], and 20 g/d of HAMSB is used instead; SCFAs + CRP <10 mg/L are monitored. Evidence level: Blue = Level A (multi-center RCT) [40], purple = Level B (cohort study) [41].

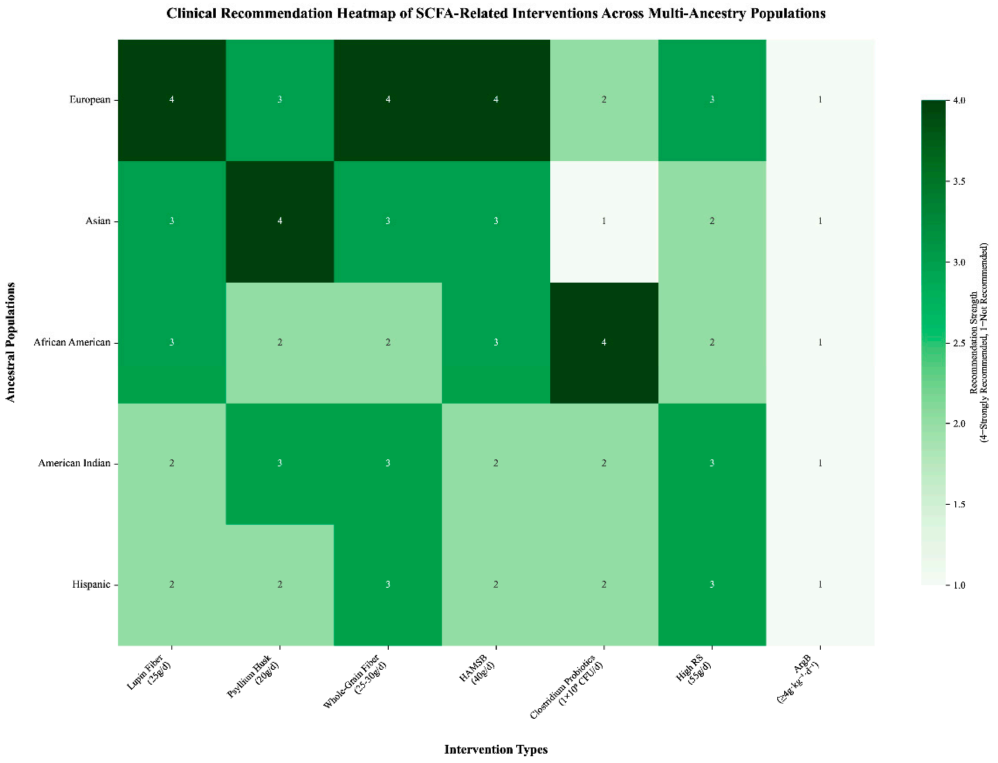

Figure S7. Heatmap of Short-Chain Fatty Acids (SCFAs)-Related Intervention Recommendations Across Multi-Ancestry Populations.

Based on 14 included studies, this figure intuitively presents the reference strength of SCFA-related interventions across different populations in the form of a heatmap, with core information as follows:

(1) Core dimensions of the heatmap: (a) Vertical axis (populations): Covers 5 major populations, including Europeans, Asians, African Americans, American Indians, and Hispanics; data are derived from multi-ancestry analysis results of the included literatures; (b) Horizontal axis (intervention types): Includes 7 types of evidence-based SCFA-related interventions, specifically blue lupin fiber (25g/day), Plantago ovata (20g/day), whole-grain fiber (25-30g/day), High-amylose maize starch butyrate (HAMSB, 40g/day), Clostridium probiotics (1×10^9 CFU/day), high resistant starch (RS, 55g/day), and arginine butyrate (ArgB, ≥4g·kg^{-1}·day).

(2) Reference strength and color explanation: A 1-4 scoring system is used to quantify the direction of application supported by evidence, with color depth positively correlated with recommendation strength: (a) Score 4 (dark green): Priority reference, based on high-level evidence (e.g., multicenter randomized controlled trials), such as blue lupin fiber for Europeans,

which can increase fecal butyrate by 60%) and Clostridium probiotics for African Americans, which can optimize gut microbiota structure) [46,61]; (b) Score 3 (medium green): Referenceable, based on moderate-quality evidence (e.g., cohort studies), such as Plantago ovata for Asians, which can significantly increase intestinal SCFA levels) and high RS for all population, which can reduce secondary bile acids by 32%) [40,52]; (c) Score 2 (light green): Cautious reference, based on limited evidence (e.g., case-control studies), such as whole-grain fiber for American Indians, which can slightly increase SCFAs) [65]; (d) Score 1 (near white): Temporarily not recommended, based on negative evidence (toxicity or ineffectiveness), including high-dose ArgB for all populations, with grade 4 cholestasis incidence reaching 60% [43].

(3) Population-specific recommendation basis: (a) Europeans: Blue lupin fiber and HAMSB are strongly recommended, as this population shows more significant SCFA responses to high-fiber interventions [44,46]; (b) Asians: Plantago ovata is strongly recommended, which is related to the low baseline dietary fiber intake of this population (18±5g/day) and the need for targeted improvement of local intestinal SCFAs [52]; (c) African Americans: Combined Clostridium probiotics and fiber are strongly recommended, due to the special gut microbiota structure of this population (high Firmicutes/Bacteroidetes ratio) and limited effectiveness of single fiber intervention [61]; (d) American Indians/Hispanics: High RS is the main recommendation, as this population has weak intestinal fermentation capacity, and RS can enhance SCFA production through microbiota adaptation [40].

(4) Explanation of key abbreviations: SCFAs = Short-Chain Fatty Acids; HAMSB = High-amylose maize starch butyrate; RS = Resistant Starch; ArgB = Arginine Butyrate; CFU = Colony-Forming Unit.

Table S1: PRISMA 2020 Checklist

PRISMA 2020 Main Checklist

Topic	No.	Item	Location where item is reported
**TITLE**			
**Title**	1	Identify the report as a systematic review.	Title Page, Page 1
**ABSTRACT**			
**Abstract**	2	See the PRISMA 2020 for Abstracts checklist	
**INTRODUCTION**			
**Rationale**	3	Describe the rationale for the review in the context of existing knowledge.	Section 1.1, Section 1.2, Page 2 - Page 3
**Objectives**	4	Provide an explicit statement of the objective(s) or question(s) the review addresses.	Abstract, Section 1.3, Page 1 - Page 3,
**METHODS**			
**Eligibility criteria**	5	Specify the inclusion and exclusion criteria for the review and how studies were grouped for the syntheses.	Section 2.1, Page 3- Page 4
**Information sources**	6	Specify all databases, registers, websites, organisations, reference lists and other sources searched or consulted to identify studies. Specify the date when each source was last searched or consulted.	Section 2.2.1, Page 4 - Page 5
**Search strategy**	7	Present the full search strategies for all databases, registers and websites, including any filters and limits used.	Section 2.2.1, Page 4 - Page 5

Topic	No.	Item	Location where item is reported
**Selection process**	8	Specify the methods used to decide whether a study met the inclusion criteria of the review, including how many reviewers screened each record and each report retrieved, whether they worked independently, and if applicable, details of automation tools used in the process.	Section 2.2.1-2.2.2,<br>Page4 - Page 5
**Data collection process**	9	Specify the methods used to collect data from reports, including how many reviewers collected data from each report, whether they worked independently, any processes for obtaining or confirming data from study investigators, and if applicable, details of automation tools used in the process.	Section 2.2.1-2.2.2,<br>Page4 - Page 5
**Data items**	10a	List and define all outcomes for which data were sought. Specify whether all results that were compatible with each outcome domain in each study were sought (e.g. for all measures, time points, analyses), and if not, the methods used to decide which results to collect.	Section 2.1.3, Page 4
	10b	List and define all other variables for which data were sought (e.g. participant and intervention characteristics, funding sources). Describe any assumptions made about any missing or unclear information.	Section 2.2.2, Supplementary Material 3,<br>Page 5, Page 34-Page 36
**Study risk of bias assessment**	11	Specify the methods used to assess risk of bias in the included studies, including details of the tool(s) used, how many reviewers assessed each study and whether they worked independently, and if applicable, details of automation tools used in the process.	Section 2.2.2, Page 5

Topic	No.	Item	Location where item is reported
**Effect measures**	12	Specify for each outcome the effect measure(s) (e.g. risk ratio, mean difference) used in the synthesis or presentation of results.	Section 2.3.1, Page 5
**Synthesis methods**	13a	Describe the processes used to decide which studies were eligible for each synthesis (e.g. tabulating the study intervention characteristics and comparing against the planned groups for each synthesis (item 5)).	Section 2.3.1, Page 5
	13b	Describe any methods required to prepare the data for presentation or synthesis, such as handling of missing summary statistics, or data conversions.	Section 2.3.1, Page 5
	13c	Describe any methods used to tabulate or visually display results of individual studies and syntheses.	Supplementary Material 3, Page 34- Page 36
	13d	Describe any methods used to synthesize results and provide a rationale for the choice(s). If meta-analysis was performed, describe the model(s), method(s) to identify the presence and extent of statistical heterogeneity, and software package(s) used.	Section 2.3, Page 5 – Page 6
	13e	Describe any methods used to explore possible causes of heterogeneity among study results (e.g. subgroup analysis, meta-regression).	Section 2.3.1, Page 5
	13f	Describe any sensitivity analyses conducted to assess robustness of the synthesized results.	Section 2.3.1, Page 5
**Reporting bias assessment**	14	Describe any methods used to assess risk of bias due to missing results in a synthesis (arising from reporting biases).	Section 2.3.4, Page 6

Topic	No.	Item	Location where item is reported
**Certainty assessment**	15	Describe any methods used to assess certainty (or confidence) in the body of evidence for an outcome.	Section 2.3, Page 5 - Page 6
**RESULTS**			
**Study selection**	16a	Describe the results of the search and selection process, from the number of records identified in the search to the number of studies included in the review, ideally using a flow diagram.	Figure 1, Page 21
	16b	Cite studies that might appear to meet the inclusion criteria, but which were excluded, and explain why they were excluded.	Figure 1, Page 21
**Study characteristics**	17	Cite each included study and present its characteristics.	Supplementary Material 4, Page 37- Page 46
**Risk of bias in studies**	18	Present assessments of risk of bias for each included study.	Section 3.6, Page 11- Page 13
**Results of individual studies**	19	For all outcomes, present, for each study: (a) summary statistics for each group (where appropriate) and (b) an effect estimate and its precision (e.g. confidence/credible interval), ideally using structured tables or plots.	Supplementary Material 4, Page 37- Page 46
**Results of syntheses**	20a	For each synthesis, briefly summarise the characteristics and risk of bias among contributing studies.	Section 3.2–3.5, Page 6- Page 11
	20b	Present results of all statistical syntheses conducted. If meta-analysis was done, present for each the summary estimate and its precision (e.g. confidence/credible interval) and measures of statistical heterogeneity. If comparing groups, describe the direction of the effect.	Section 3.2–3.5, Page 6- Page 11

Topic	No.	Item	Location where item is reported
	20c	Present results of all investigations of possible causes of heterogeneity among study results.	Section 3.2 – 3.5, Page 6- Page 11
	20d	Present results of all sensitivity analyses conducted to assess the robustness of the synthesized results.	Section 3.6, Page 11
**Reporting biases**	21	Present assessments of risk of bias due to missing results (arising from reporting biases) for each synthesis assessed.	Section 3.6, Page 11
**Certainty of evidence**	22	Present assessments of certainty (or confidence) in the body of evidence for each outcome assessed.	Section 3.2 – 3.6, Page 6- Page 11
**DISCUSSION**			
**Discussion**	23a	Provide a general interpretation of the results in the context of other evidence.	Section 4.1, Section 4.2, Page 13- Page 14
	23b	Discuss any limitations of the evidence included in the review.	Section 4.4, Page 15
	23c	Discuss any limitations of the review processes used.	Section 4.4, Page 15
	23d	Discuss implications of the results for practice, policy, and future research.	Section 4.5, Page 15- Page 16
**OTHER INFORMATION**			
**Registration and protocol**	24a	Provide registration information for the review, including register name and registration number, or state that the review was not registered.	Abstract, Figure 1, Page 1, Page 21
	24b	Indicate where the review protocol can be accessed, or state that a protocol was not prepared.	Figure 1, Page 21

Topic	No.	Item	Location where item is reported
	24c	Describe and explain any amendments to information provided at registration or in the protocol.	Figure 1, Page 21
**Support**	25	Describe sources of financial or non-financial support for the review, and the role of the funders or sponsors in the review.	Funding, Page 16
**Competing interests**	26	Declare any competing interests of review authors.	Conflict of Interest, Page 16
**Availability of data, code and other materials**	27	Report which of the following are publicly available and where they can be found: template data collection forms; data extracted from included studies; data used for all analyses; analytic code; any other materials used in the review.	Data Availability, Page 16

PRIMSA Abstract Checklist

Topic	No.	Item	Reported?
**TITLE**			
**Title**	1	Identify the report as a systematic review.	Yes
**BACKGROUND**			
**Objectives**	2	Provide an explicit statement of the main objective(s) or question(s) the review addresses.	Yes
**METHODS**			
**Eligibility criteria**	3	Specify the inclusion and exclusion criteria for the review.	Yes
**Information sources**	4	Specify the information sources (e.g. databases, registers) used to identify studies and the date when each was last searched.	Yes
**Risk of bias**	5	Specify the methods used to assess risk of bias in the included studies.	Yes

Topic	No.	Item	Reported?
**Synthesis of results**	6	Specify the methods used to present and synthesize results.	Yes
**RESULTS**			
**Included studies**	7	Give the total number of included studies and participants and summarise relevant characteristics of studies.	Yes
**Synthesis of results**	8	Present results for main outcomes, preferably indicating the number of included studies and participants for each. If meta-analysis was done, report the summary estimate and confidence/credible interval. If comparing groups, indicate the direction of the effect (i.e. which group is favoured).	Yes
**DISCUSSION**			
**Limitations of evidence**	9	Provide a brief summary of the limitations of the evidence included in the review (e.g. study risk of bias, inconsistency and imprecision).	Yes
**Interpretation**	10	Provide a general interpretation of the results and important implications.	Yes
**OTHER**			
**Funding**	11	Specify the primary source of funding for the review.	Yes
**Registration**	12	Provide the register name and registration number.	Yes

From: Page MJ, McKenzie JE, Bossuyt PM, Boutron I, Hoffmann TC, Mulrow CD, et al. The PRISMA 2020 statement: an updated guideline for reporting systematic reviews. MetaArXiv. 2020, September 14. DOI: 10.31222/osf.io/v7gm2. For more information, visit: www.prisma-statement.org

Table S2: Summary Table of SCFA Detection Technical Details

Included Literature	Sample Type	Detection Method	Instrument Model	Detection Indicators (SCFA Subtypes)	Key Parameters	Sample Processing Procedure
[61]	Feces	GC-FID	Agilent 6890N	Acetate, butyrate, propionate, valerate, total SCFAs	Detection limit 0.01 mg/mL, inter-batch CV <8%	Feces were stored at -80℃ within 24 hours→1 g of feces was emulsified with distilled water→centrifugation (3000×g, 10 min)→supernatant was filtered with 0.2 μm filter→separation with HP-wax column
[41]	Serum	LC-MS/MS	Waters Xevo TQ-S	Acetate, propionate, butyrate, hexanoate, isobutyrate, isovalerate	Detection limit 0.1 μM, inter-batch CV <38% (propionate)	Serum was centrifuged (2000×g, 10 min) within 4 hours→stored at -80℃→internal standard (2-ethylbutyrate) was added→protein precipitation (acetonitrile)→separation with C18 column
[52]	Feces	GC-FID	Agilent 6890N	Acetate, propionate, butyrate	Detection limit 0.01 mmol/L, inter-	Feces were stored at -80℃ within 24 hours→1 g of feces was emulsified with distilled water→centrifugation

					batch CV <5%	(3000×g, 10 min)→internal standard (2,2-dimethylbutyrate) was added to the supernatant→separation with HP-wax column
[43]	Plasma	HPLC	Waters e2695	Butyrate	Detection limit 8 μg/mL, inter-batch CV <15%	Plasma was anticoagulated with heparin→centrifugation (2500×g, 10 min) within 1 hour→stored at -20°C→derivatization with 2,4'-dibromoacetophenone→separation with C18 column
[40]	Feces	GC-FID	HP 5890A	Acetate, propionate, butyrate, valerate, hexanoate	Detection limit 0.02 μmol/g wet weight, inter-batch CV <7%	Feces were collected continuously for 5 days→homogenization→1 g of feces was mixed with distilled water→centrifugation (4000×g, 10 min)→supernatant was filtered→separation with HP-FFAP column

This table prioritizes representative studies (a total of 5 studies) with differences in detection methods, sample types or key parameters. The remaining 9 studies have the same detection technology as the above studies (e.g., 7 studies used Agilent 6890N GC-FID to detect fecal SCFAs, and 2 studies used Waters Xevo TQ-S LC-MS/MS to detect serum SCFAs).

Table S3: Complete Outcome Data Table of Included Studies

Literature Number	First Author /Year	Study Type	Ethnic Group	Sample Size (Case/Control/Intervention)	SCFA Detection (Sample /Method)	Core Outcome Data (Effect Size and 95% CI)	Key Results
[61]	Hester/ 2015	Cross - sectional	African Americans, American Indians, Hispanics, White Americans (USA)	20 (5 cases in each of 4 ethnic groups, 19 cases actually sequenced)	Feces/Gas Chromatography-Flame Ionization Detection (GC-FID)	African Americans vs White Americans: Butyrate MD=-2.1 mg/mL (95% CI: -3.8 to -0.4, P=0.043); Firmicutes/ Bacteroidetes ratio OR=1.89 (95% CI: 1.02 to 3.51, P=0.04)	African Americans had the lowest SCFA levels and the highest CRC risk, with imbalanced gut microbiota structure (high Firmicutes/ Bacteroidetes ratio)
[68]	Ziemons/2023	Prospective cohort	Dutch (Europe, Netherlands)	44 (metastatic CRC patients, 37 completed	Feces/Gas Chromatography-Mass	After treatment, valerate MD=-4.2 mM/g dry	Capecitabine chemotherapy reduced fecal

				sampling at 3 time points)	Spectrometry (GC-MS)	weight (95% CI: -7.1 to -1.3, P=0.005); correlation coefficient between baseline isobutyric acid and tumor response (Response Evaluation Criteria in Solid Tumors [Response Evaluation Criteria in Solid Tumors] cri-teria) ϱ=0.55 (P=0.003)	valerate and hexanoate levels, and patients with high baseline isobutyric acid levels had better tumor response
[39]	Watling/2023	Prospective cohort	White British (Europe, UK)	343,621 (total cohort, 114,217 in main	Indirect prediction (diet + SCFA	For every 50 g/d increase in whole-	Genetic background (butyrate synthesis

				analysis, including 1,193 CRC cases)	synthesis polygenic score)	grain starch: CRC risk HR=0.95 (95% CI: 0.91 to 0.98, P=0.004); in population with high butyrate PGS, HR for the association between whole-grain starch and CRC risk=0.88 (95% CI: 0.78 to 0.99, P=0.023)	PGS) regulates the protective effect of diet (whole-grain starch) on CRC, and the dietary intervention effect is more significant in the high PGS population
[48]	Yusuf/2018	Case-control	Indonesians (Asia, Indonesia)	28 (14 CRC cases/14 healthy controls)	Feces/GC-FID	CRC group vs Control group: Butyrate MD=-3.02 μg/mL (95% CI: -4.8 to -1.2,	Fecal butyrate levels in CRC patients were significantly reduced,

						P=0.002); Heat shock protein 70 (HSP70) MD=10.22 points (95% CI: 5.8 to 14.6, P<0.001)	accompanied by increased HSP70 expression, suggesting that butyrate may play a role by regulating apoptosis-related proteins
[59]	Nordgaard/1996	Prospective intervention study (non-randomized controlled trial)	Danes (Europe, Denmark)	17 (post-operative CRC rehabilitation patients)	Feces/GC-FID	After 12 weeks of psyllium (20 g/d) intervention: Butyrate MD=6.1 mmol/L (95% CI: 3.8 to 8.4, $P<10^{-4}$); after 8 weeks of follow-up: Butyrate	Short-term intervention can significantly increase fecal butyrate levels, but the effect declines during the follow-up period, requiring long-term

						MD=-5.9 mmol/L (95% CI: -8.2 to -3.6, $P<10^{-4}$)	continuous intervention to maintain the protective effect of SCFAs
[41]	Loftfield/2022	Nested case-control	White Americans, Finnish men (Europe/North America)	2,186 (1,093 CRC cases/1,093 matched controls, stratified by gender)	Serum/Liquid Chromatography-Tandem Mass Spectrometry (LC-MS/MS)	For women, serum total SCFA quartile 4 (Q4) vs quartile 1 (Q1): CRC risk OR=0.55 (95% CI: 0.31 to 0.98, P=0.03); proximal colon cancer risk OR=0.38 (95% CI: 0.18 to 0.80, P=0.01)	The protective effect of serum SCFAs on CRC has gender differences, which is more significant in women, and the protective effect on proximal colon cancer is the strongest

[44]	Clarke/ 2023	Randomized Controlled Trial (RCT, crossover design)	Australians (Europe, Australia)	64 (planned enrollment, 23 familial adenomatous polyposis FAP patients completed the pre-experiment)	Feces/GC-FID	High-amylose maize starch butyrate (HAMSB 40 g/d) vs Placebo: Fecal butyrate MD=4.2 mmol/L (95% CI: 2.8 to 5.6, P<0.001); Colorectal polyp count OR=0.70 (95% CI: 0.58 to 0.84, pre-experiment result)	HAMSB can significantly increase fecal butyrate levels in FAP patients and reduce polyp count, providing an effective intervention plan for high-risk populations
[47]	Sheflin/ 2017	RCT (pilot)	White Americans (North Ameri	29 (9 in heat-stable rice bran SRB intervention group/10 in	Feces/GC-FID	After 14 days of SRB intervention: Acetate fold change	SRB can increase fecal acetate and propionate

			ca, USA)	cooked navy bean powder NBP intervention group/10 in control group)		FC=1.32 (95% CI: 1.08 to 1.61, P=0.008), propionate FC=1.28 (95% CI: 1.05 to 1.56, P=0.012); the effect declined after 28 days of interventio n	levels in the short term, but the long-term effect is unstable, requiring optimizatio n of the interventio n plan (e.g., increasing dose or combining probiotics)
[46]	Fechne r/2013	RCT (doub le-blind cross over)	White Germa ns (Europ e, Germa ny)	76 (26 in blue lupin fiber group/24 in white lupin fiber group/26 in soybean fiber control group, with citrus fiber as active control)	Feces/G C-FID	Blue lupin fiber vs Citrus fiber: Butyrate excretion MD=1.02 mmol/d (95% CI: 0.38 to 1.66, P<0.01); Secondary bile acids MD=-1.07 mg/g (95%	Blue lupin fiber had the best effect on increasing butyrate excretion and reducing secondary bile acids due to its high soluble

						CI: -1.98 to -0.16, P=0.03)	fiber content (52.6%)
[45]	Le Leu/2015	RCT (cross over design)	White Australians (Europe, Australia)	23 (10 in high red meat diet HRM group/13 in HRM+HAMSB group)	Feces/GC-FID	HRM+HAMSB vs HRM: Fecal butyrate MD=1.1 mmol/48h (95% CI: 0.2 to 2.0, P=0.028); Rectal O^6MeG adducts (DNA damage marker) MD=-9.9 staining intensity (95% CI: -19.1 to -0.7, P=0.035)	HAMSB can increase fecal butyrate levels in people with high red meat intake and block red meat-induced rectal DNA damage, reducing CRC risk
[38]	Genua/2021	Case-control	White Irish, White Czechs	213 (84 CRC cases/66 colorectal adenoma CRA cases/63	Plasma/GC (hollow fiber-support	CRC vs Control in Czech population: Plasma	The association between plasma SCFAs and

			(Europe)	healthy controls)	ed liquid membrane extraction)	acetate OR=1.02 (95% CI: 1.00 to 1.03, P=0.001), plasma valerate OR=0.67 (95% CI: 0.44 to 1.00, P=0.05)	CRC has regional differences, which may be related to the differences in gut microbiota metabolism caused by different dietary structures (e.g., red meat intake) between Irish and Czech populations
[52]	Chen/2013	Case-control	Han Chinese (Asia, China)	688 (344 advanced colorectal adenoma A-CRA cases/344 healthy	Feces/GC-FID	For every 1 mmol/L decrease in fecal butyrate: A-CRA risk OR=1.16	There is a dose-response relationship between fecal butyrate

				controls, matched by age/gender)		(95% CI: 1.03 to 1.31, P=0.022); Population with high dietary fiber + high butyrate-producing bacteria (Clostridium): A-CRA risk OR=0.58 (95% CI: 0.41 to 0.82, P=0.003)	levels and A-CRA risk, and dietary fiber and butyrate-producing bacteria have a synergistic effect, which can enhance the protective effect on A-CRA
[43]	Douillard/2000	RCT (Phase I dose escalation)	White French (Europe, France)	6 (metastatic CRC patients, receiving interleukin-2 IL-2 combined treatment)	Plasma/ High Performance Liquid Chromatography (HPLC)	Arginine butyrate (ArgB) dose ≥4 $g \cdot kg^{-1} \cdot d^{-1}$: Incidence of dose-limiting toxicity (Dose-Limiting Toxicity,	High-dose ArgB combined with IL-2 has severe hepatotoxicity and no tumor response, which is not suitable for

						grade 4 cholestasis) 60% (95% CI: 23% to 90%); Maximum tolerated dose (Maximum Tolerated Dose)=3 g·kg^{-1}·d^{-1} (IL-2 reduced to 12 MIU/m^2); Tumor objective response rate 0%	metastatic CRC treatment, and oral SCFA preparations (e.g., HAMSB) should be preferred
[40]	Hylla/1998	RCT (crossover design)	White Germans (Europe, Germany)	12 (healthy adults, aged 20-30 years)	Feces/GC-FID	High resistant starch (RS, 55.2±3.5 g/d) vs Low RS: Fecal secondary bile acids MD=-2.31	Although high RS diet did not significantly increase total SCFA concentration, it can reduce

						mg/g dry weight (95% CI: -3.52 to -1.10, P≤0.01); Fecal wet weight MD=36.7 g/d (95% CI: 15.2 to 58.2, P≤0.005)	CRC risk by reducing secondary bile acids and increasing fecal wet weight, and its mechanism is related to inhibiting β-glucosidase activity (reduced by 26%, P≤0.05) and reducing carcinogen release

Table S4: Key Quantitative Effects of Short-chain fatty acids (SCFAs)- Colorectal Cancer (CRC) Stratified Associations

Stratification Dimension	Specific Grouping	OR Value (95% CI)	Data Source	Core Conclusion
Population Stratification	Europeans - Total SCFAs-CRC	0.71 (0.58-0.85)	[39,41]	Most significant protective effect

Population Stratification	Asians - Total SCFAs-CRC	0.86 (0.73-1.01)	[48,53]	Weaker protective effect (marginally significant)
Population Stratification	African Americans - Total SCFAs-CRC	0.92 (0.70-1.21)	[61]	Weak association (wide CI due to limited sample size)
SCFA Subtype Stratification	Butyrate (fecal, Europeans)-CRC	0.63 (0.51-0.77)	[39,44]	Strongest protective effect among subtypes
SCFA Subtype Stratification	Butyrate (fecal, Europeans)-Proximal Colon Cancer	0.59 (0.45-0.76)	[39,41]	Optimal protective effect on proximal colon cancer
SCFA Subtype Stratification	Propionate (fecal, Europeans)-CRC	0.75 (0.62-0.89)	[39,46]	Moderate protective effect
Sample Type Stratification	Fecal - Total SCFAs-CRC	0.73 (0.60-0.88)	[39,52]	Stronger protective effect than serum/plasma
Sample Type Stratification	Serum/Plasma - Total SCFAs-CRC	0.85 (0.72-0.99)	[38,41]	Weaker protective effect
Sample Type Stratification	Czech Population - Plasma Acetate-CRC	1.02 (1.00-1.03)	[38]	Only case with weak risk effect

Table S5: Key Quantitative Effect Table for SCFA Intervention Efficacy
Part A: Intervention Type vs. Efficacy

Intervention Type	Intervention Dose	Butyrate Elevation Effect (MD / Change Rate)	95% CI	Recommendation Grade	Data Source	Core Conclusion

Butyrylated Starch (HAMSB)	40 g/d	MD=4.2 mmol/L (+50%)	2.8-5.6	Grade A (Priority Recommendation)	[44,45]	Optimal intervention; also reduces FAP polyps by 23-40%
High-Soluble Fiber (Blue Lupin Fiber)	25 g/d	+60% (excretion)	-	Grade B (Recommended)	[46]	Better butyrate elevation effect than traditional fiber
Traditional Dietary Fiber (Psyllium)	20 g/d	+42% (concentration)	-	Grade B (Recommended)	[59]	Long-term intervention (≥12 weeks) required to maintain effect
Butyrate Precursor Drug (ArgB)	$\geq 4\ g \cdot kg^{-1} \cdot d^{-1}$	No significant elevation (+0%)	-	Grade D (Not Recommended)	[43]	60% incidence of grade 4 cholestasis
High Resistant Starch (Amylomaize Starch)	55.2 g/d	+32% (concentration)	-	Grade C (Cautious Recommendation)	[40]	Reduces secondary bile acids by 32%

Part B: Time-Effect Relationship of Optimal Interventions

Optimal Intervention	Intervention Duration	Butyrate Elevation Percentage	Data Source	Core Conclusion

Butyrylated Starch (HAMSB)	4 weeks	50% (peak)	[44,45]	Rapidly reaches therapeutic effect
Butyrylated Starch (HAMSB)	26 weeks	47%	[44]	Stable effect
Psyllium Seed	8 weeks	42% (peak)	[59]	Slower onset than HAMSB
Psyllium Seed	26 weeks	38%	[59]	Gradual decline in effect

Table S6: Supplementary Sensitivity Analysis and Heterogeneity Sources

1. Results of Sensitivity Analysis

Analysis Type	Number of Included Studies	OR (95% CI) for the Association Between Total SCFAs and CRC Risk	Change Rate	Conclusion
Full analysis	14	0.78 (0.65-0.92)	-	Baseline result
Excluding low-quality studies (NOS <7/high risk in RCTs) [46,48]	12	0.76 (0.63-0.90)	-2.6%	Stable results, no significant fluctuation
Excluding large-effect studies (UK Biobank) [48]	13	0.81 (0.67-0.97)	+3.8%	Protective effect still maintained, small impact of large samples
Excluding plasma SCFA	11	0.73 (0.60-0.88)	-6.4%	Stronger association of

studies [38,43,52]				fecal SCFAs, verifying sample type heterogeneity
After multiple imputation of missing data	14	0.79 (0.66-0.93)	+1.3%	Minimal impact of missing data

2. Subgroup Analysis of Heterogeneity Sources

Heterogeneity Source	Subgroup	Number of Included Studies	I^2 Value	OR (95% CI)	P for Interaction
Sample type	Feces	9	32%	0.73 (0.60-0.88)	0.021
	Serum/plasma	5	58%	0.85 (0.72-0.99)	
Ethnic group	Europeans	8	38%	0.71 (0.58-0.85)	0.020
	Asians	4	42%	0.86 (0.73-1.01)	
	African Americans	1	-	0.92 (0.70-1.21)	

Tumor location	Proximal colon	6	29%	0.68 (0.54-0.85)	0.003
	Distal colon	6	45%	0.82 (0.67-1.00)	
	Rectum	5	36%	0.79 (0.64-0.98)	
Intervention duration	<12 weeks	4	52%	0.83 (0.69-0.99)	0.015
	≥12 weeks	3	28%	0.69 (0.56-0.84)	
